# Tonotopic organization in the basal region of the ventromedial nucleus of the thalamus revealed by fiber photometry recording

**DOI:** 10.1117/1.NPh.13.1.015008

**Published:** 2026-01-29

**Authors:** Mahiber Polat, Jie Tao, Yiheng Chen, Sunny C. Li, Zhikai Zhao, Xiaowei Chen

**Affiliations:** a Chongqing University, College of Bioengineering, Chongqing, China; b LFC Laboratory and Chongqing Institute for Brain and Intelligence, Guangyang Bay Laboratory, Chongqing, China; c Guangxi University, School of Medicine, Advanced Institute for Brain and Intelligence, Nanning, China; d Third Military Medical University, Brain Research Center and State Key Laboratory of Trauma and Chemical Poisoning, Chongqing, China; e Newlight Neuroscience Unit, Chongqing, China; f Chongqing University, School of Medicine, Center for Neurointelligence, Chongqing, China

**Keywords:** tonotopy, auditory thalamus, fiber photometry, ventromedial thalamus, fine discrimination, Ca^2+^ recording

## Abstract

**Significance:**

Although the transmission of auditory information in the brain has been extensively studied, the mechanism underlying fine auditory discrimination remains incompletely understood. The basal region of the ventromedial nucleus of the thalamus (bVM) has recently been found to convey frequency-specific auditory information to the primary auditory cortex (A1). Inhibition of the bVM significantly impairs fine auditory discrimination in mice. These findings indicate that the bVM plays an important role in frequency information processing. However, direct functional evidence for tonotopic organization within the bVM is still lacking.

**Aim:**

We aimed to investigate whether bVM neurons exhibit a spatially ordered frequency preference, using a simple yet efficient *in vivo* functional mapping strategy.

**Approach:**

To characterize the response properties of bVM neurons projecting to A1, we combined Cre-dependent retrograde viral labeling with fiber photometry in awake mice. Using a “one recording site per animal” strategy, we systematically recorded from 26 distinct locations and successfully reconstructed the tonotopic map of the bVM.

**Results:**

We identified a mediolateral tonotopic gradient within the bVM, with best frequencies progressing from low in medial regions to high in lateral regions.

**Conclusions:**

Our findings provide direct functional evidence of tonotopic organization within the bVM, supporting its role as an auditory relay and its contribution to fine auditory discrimination.

## 1 Introduction

The central nervous system processes sensory inputs through functionally specialized and spatially segregated circuits, enabling selective feature extraction and integration to construct coherent perceptions.^1^^,^^2^ In the visual system, this specialization is exemplified by orientation columns and ocular dominance columns.^3^^,^^4^ The auditory system employs a tonotopic organization wherein neurons are systematically arranged according to their frequency preferences.^5^ This frequency-based spatial arrangement, known as tonotopy, is highly conserved across vertebrates, ranging from fish to mammals.^6^^,^^7^ Tonotopy has been thought to underlie various core auditory functions, including frequency-specific signal transmission,^5^ spectrotemporal integration,^8^ and hierarchical feature extraction.^9^ Disruption of the tonotopic map is associated with several auditory disorders. For instance, noise-trauma-induced tinnitus is accompanied by reorganization of the tonotopic map.^10^^,^^11^ Distorted frequency mapping following acoustic injury impairs noise-robust speech representation in auditory pathways.^12^

In mammals, this conserved tonotopic organization is preserved along a well-defined transmission pathway. First, the cochlea establishes a frequency-based arrangement where hair cells arrayed along the basilar membrane are spatially tuned to distinct frequencies, forming an orderly tonotopic map.^13^ This mechanical map is transduced into neural signals within the cochlea and is subsequently relayed and processed through the cochlear nucleus,^14^^,^^15^ the lateral superior olive,^16^ the central nucleus of the inferior colliculus (CIC),^17^ and ultimately via the ventral division of the medial geniculate body (MGBv) to the primary auditory cortex (A1), where a highly refined tonotopic representation emerges.^18^^,^^19^ As the principal auditory thalamic relay, the MGBv plays a critical dual role in the transmission and refinement of the tonotopic map. First, it faithfully maintains and relays frequency-specific information from lower levels, ensuring the reliable projection of the tonotopic architecture to A1. More importantly, the MGBv receives feedback signals from layer VI of the auditory cortex (AuC) to sharpen tuning curves, thereby playing a crucial role in auditory perception and scene analysis.^20^^–^^22^

Beyond the canonical auditory pathway, emerging evidence suggests the presence of weak or non-canonical tonotopic organization in other brain regions. For instance, the insular auditory field, located rostral to the AuC, displays a rostroventral-to-dorsocaudal tonotopic gradient in response to pure tones and receives inputs from the MGBv.^23^^,^^24^ In another study, striatal neurons receiving inputs from A1 exhibited frequency-dependent potentiation during reward-associated auditory discrimination tasks. Complementary *in vitro* brain slice experiments further revealed distinct spatially segregated responses to different frequencies.^25^ Collectively, these findings demonstrate that auditory information processing is not confined to the classical ascending pathway but involves a broader range of subcortical and cortical circuits, which may utilize spatially organized frequency representations to support auditory functions. In auditory neuroscience, it remains an open question whether other less characterized nuclei also exhibit well-organized tonotopic maps.

Our previous work identified a tonotopically organized excitatory projection from the basal region of the ventromedial nucleus of the thalamus (bVM) to A1.^26^ Retrograde tracing combined with functional mapping of A1 suggested that bVM neurons are likely arranged in a tonotopic manner to mirror this projection. However, this inference relied primarily on anatomical projection. Direct functional evidence confirming tonotopic organization among bVM neurons is still lacking. Elucidating this intrinsic organization is essential for understanding the mechanistic contribution of this nucleus to auditory processing and, ultimately, to frequency discrimination. Although the canonical auditory thalamus, medial geniculate body (MGB), resides on the posterolateral thalamic surface (medial to the hippocampus),^27^ the bVM is located in the basal region of the ventromedial thalamic nucleus.^26^^,^^28^ Given the deep location of the bVM, technical challenges arise in functional mapping.

Methodologically, mapping the organization of tonotopy in deep brain structures faces trade-offs among resolution, specificity, invasiveness, and depth. Electrophysiological techniques such as neuropixels^29^ offer unparalleled temporal resolution for stimulus-evoked responses at the single-neuron level, yet they are limited in their ability to selectively target genetically defined cell populations. Wide-field ${\mathrm{Ca}}^{2+}$ imaging provides direct visualization of mesoscale signals across superficial cortical areas,^19^^,^^30^ but its application is intrinsically limited to superficial structures due to light scattering constraints in deep subcortical nuclei. Although two-photon ${\mathrm{Ca}}^{2+}$ imaging provides cellular-resolution access to defined neuronal populations in deep brain structures,^31^ this approach requires the implantation of gradient refractive index (GRIN) lenses and faces several challenges. First, the implantation procedure entails significant surgical risks, including postoperative tissue deformation and potential damage to other critical nuclei. Second, optical aberrations in GRIN lenses cause non-uniform image quality across the field of view (FOV) and distort the apparent locations of neurons.^32^^,^^33^ These constraints collectively limit high-fidelity recording of tonotopic organization in deep brain regions such as the bVM. Optrodes can achieve single-cell resolution from genetically targeted neurons but face the spatial constraints of the fiber and two major technical complexities: (1) the requirement for precise spatial alignment between the optical fiber and the electrode array to ensure targeted optogenetic stimulation alongside reliable electrophysiological sampling and (2) the necessity of mitigating photoelectric artifacts, which often requires specialized probe geometries or *post hoc* signal correction.^34^ Finally, although functional magnetic resonance imaging is invaluable in humans for large-scale non-invasive mapping,^35^ its limited spatiotemporal resolution makes it less suitable for resolving fine-scale tonotopy in small animal models.

In this context, given that reconstructing the bVM tonotopic map prioritizes structural fidelity of the brain and sufficient imaging depth over cellular resolution, we consider fiber photometry more suitable than other techniques (e.g., two-photon imaging) for this study. It enables reliable recording of genetically defined neuronal population activity at targeted locations along deep brain regions such as the bVM while offering the advantages of minimal tissue deformation and loss. Although fiber photometry has a restricted FOV like other techniques, this limitation is offset by its operational simplicity, which allows for efficient implementation of a “one recording site per animal” strategy across multiple mice to achieve comprehensive bVM coverage. It thus provides an optimal balance between the achievable mapping scale and spatial resolution for this specific task.^36^^–^^38^

Here, we took advantage of fiber photometry and systematically mapped the tonotopic organization of the bVM in awake mice. By recording pure tone-evoked ${\mathrm{Ca}}^{2+}$ signals from virally labeled bVM neurons across 26 distinct recording sites in awake mice, we aimed to (1) quantitatively characterize the frequency tuning of bVM neuronal populations and (2) establish the relationship between the anatomical location of recorded neurons and their frequency preferences. Our findings provide direct evidence for a tonotopic organization within the bVM. This establishes a critical foundation for future investigations into its precise contribution to auditory perception and behavior.

## 2 Methods

### 2.1 Animals

C57BL/6J male mice (2-month-old) were obtained from the Laboratory Animal Center of the Third Military Medical University. Mice were group-housed (up to four per cage) under controlled conditions (22±1, °C, 55±5% humidity) with a 12-h light–dark cycle (lights on at 07:00 a.m.). Food and filtered water were available *ad libitum*. All procedures were approved by the Animal Ethics Committee of the Third Military Medical University and conformed to institutional guidelines for animal care and use (Animal Ethical Statement No. AMUWEC20250039).

### 2.2 Stereotaxic Viral Injection

Mice were anesthetized with 1 to 2% isoflurane in oxygen and placed on a stereotaxic apparatus. The eyes were lubricated with ophthalmic ointment (e.g., Lacri-Lube) to prevent dryness. The core body temperature was maintained at 37±0.5°C using a heating pad. For A1 targeting, a craniotomy (∼0.5-mm diameter) was made at anterior-posterior (AP) =−3.0  mm and mediolateral (ML) = 3.8 mm (relative to bregma). A glass micropipette angled 28 deg medially was lowered to a depth of dorsoventral (DV) =−1.45  mm (from the dura). After a 5-min stabilization period, 100 to 200 nL of AAV2/2Retro-hSyn-Cre (titer: $1\times 10^{13}\text{ viral particles}/\mathrm{mL}$) was pressure-injected over 5 min. To target the bVM, a second craniotomy was made at AP=−1.70 and ML = 0.7 mm. The pipette was lowered to DV=−4.40  mm, and 70 nL of AAV2/9-hSyn-DIO-GCaMP6f (titer: $5\times 10^{12}\text{ viral particles}/\mathrm{mL}$) was delivered over 10 min. After injection, the pipette was left in place for 10 min to prevent reflux. Scalp incisions were sutured using absorbable thread under aseptic conditions.

### 2.3 Fiber Photometry Setup

**Fig. 1:**
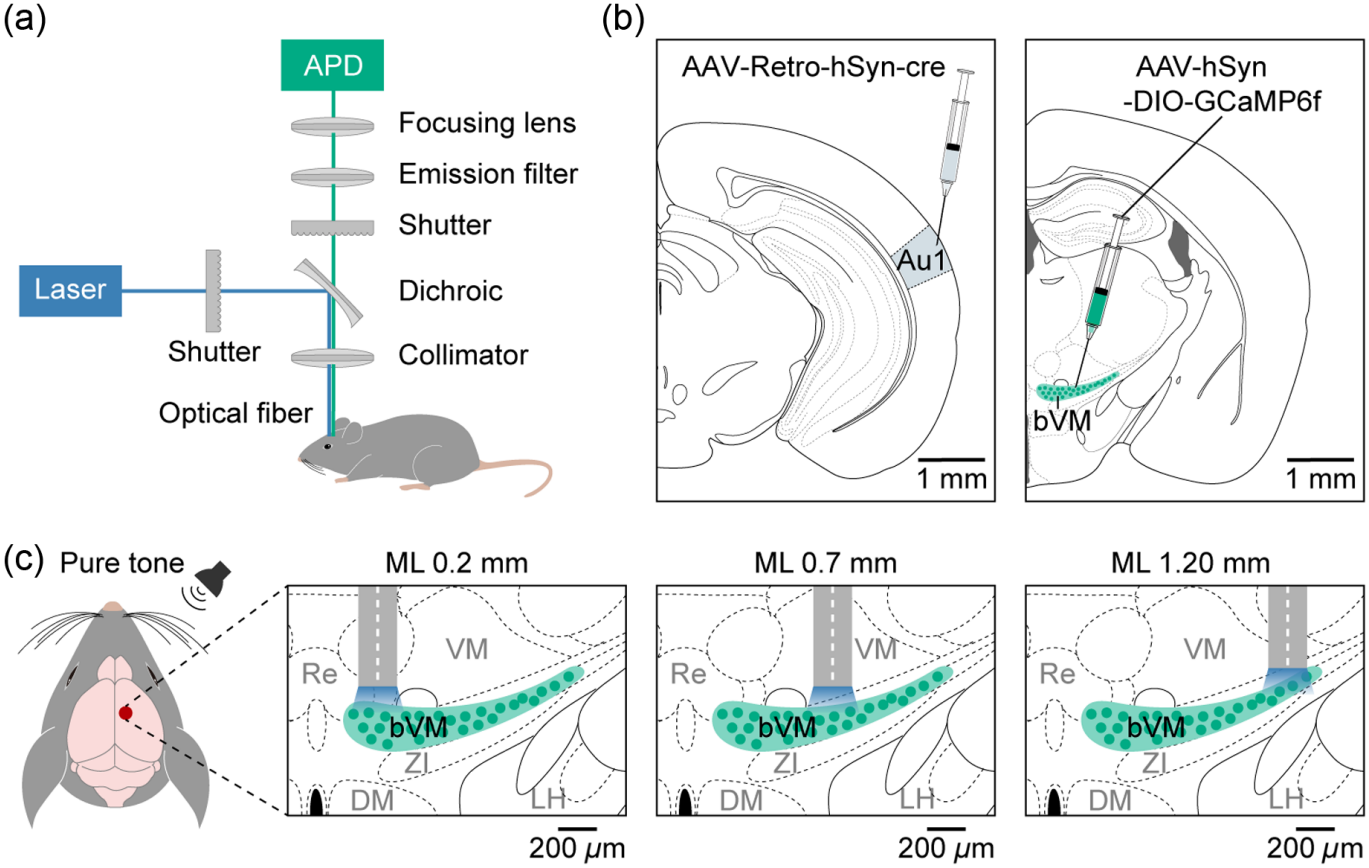
Experimental design and fiber photometry setup for ${\mathrm{Ca}}^{2+}$ recording in bVM. (a) Schematic diagram of the fiber photometry system used for recording ${\mathrm{Ca}}^{2+}$ signals from the bVM. (b)Viral labeling strategy for bVM neurons. AAV-DIO-GCaMP6f was injected into the Au1, and Retro-hSyn-Cre was injected into the bVM. (c) Experimental paradigm of pure-tone stimulation and fiber photometry recordings at different recording sites across the bVM.

The fiber photometry system was optimized for GCaMP6f signal acquisition [Fig. 1(a)]. A 488-nm laser (Sapphire, Coherent, Saxonburg, Pennsylvania, United States) delivered $0.22\;\mathrm{mW}/{\mathrm{mm}}^{2}$ at the fiber tip. The laser beam was collimated with an achromatic lens, expanded twofold with a beam expander, and focused into the optical fiber via a 10× objective. Emitted fluorescence was separated from excitation light using a dichroic mirror and bandpass filter and then detected by a high-sensitivity silicon avalanche photodiode (APD, Hamamatsu, S2382, Hamamatsu, Japan). An optical fiber (200-μm diameter, NA 0.22, Thorlabs, Newton, New Jersey, United States) was used to maximize signal collection. The chosen NA provided a balance between collection efficiency and minimization of scattering-related background. To reduce motion artifacts, the fiber was affixed to the skull using dental cement (Tetric EvoCeram, 595953WW). Data acquisition was controlled via a custom LabVIEW 2021 program (National Instruments, Austin, Texas, United States).

### 2.4 Fiber Implantation Surgery

Mice were anesthetized with 1 to 2% isoflurane in oxygen. After disinfection with povidone–iodine and 70% ethanol, the animals were secured in a stereotaxic frame using ear bars and a nose clamp. The body temperature was maintained at 37.0±0.5°C using a heating pad. A 200-μm core optical fiber was stereotaxically implanted into the bVM at AP=−1.70  mm and ML=0.7  mm. The fiber tip was positioned ∼200  μm dorsal to the target depth of DV=−4.40  mm (i.e., at DV=−4.20  mm) to account for tissue displacement during recovery. The fiber was secured using a three-stage procedure: (1) primary adhesion with light-cured dental cement around the fiber-cannula interface, (2) structural reinforcement using three stainless steel skull screws embedded in acrylic resin, and (3) final encapsulation with multiple layers of black dental cement. A recovery period of at least 1 week was allowed before the photometry recordings.

### 2.5 Auditory Stimulation

The auditory stimuli were generated via LabVIEW 2021 (National Instruments) using a PCIe-6353 DAQ card (National Instruments). Pure-tone stimuli (4 to 54 kHz, previously used^39^^,^^40^) were delivered through an ES1 free-field speaker driven by an ED1 electrostatic amplifier (Tucker Davis Technologies, Alachua, Florida, United States). Each frequency was presented 10 times with randomized inter-stimulus intervals (5 to 8 s). Each tone lasted 50 ms. Background noise was kept at a 50-dB sound pressure level (SPL).

### 2.6 Histological Verification

Following recordings, mice were deeply anesthetized with sodium pentobarbital (100 mg/kg, i.p.) and transcardially perfused with 0.9% saline followed by 4% paraformaldehyde (PFA). The brains were post-fixed overnight in 4% PFA at 4°C, and the coronal sections (100  μm thickness) were prepared using a cryostat at −20°C. The sections were mounted on Superfrost Plus slides and coverslipped with antifade mounting medium. Viral expression and fiber placements were verified under a fluorescence microscope.

### 2.7 Data Analysis

We performed ${\mathrm{Ca}}^{2+}$ signal analysis based on established methodologies from previous studies.^36^^,^^41^ To analyze the response probability, a third-order polynomial curve was fit to the data points following prior works.^42^^,^^43^ For the third-order polynomial $P(X)=\beta_{3}X^{3}+\beta_{2}X^{2}+\beta_{1}X+\beta_{0}$, where P(X) represents response probability; $X^{3}$, $X^{2}$, and X are the normalized x-coordinates; and $\beta_{3}$, $\beta_{2}$, $\beta_{1}$, and $\beta_{0}$ are the polynomial coefficients. To quantify the spatial rate of best frequency (BF) variation across different bVM locations, a local slope was computed at each recording site using a seven-site window (the target site and its three nearest neighbors on each side) and performed linear fitting on the slope values of these seven points. This analytical approach of slope is based on a previous study.^18^

### 2.8 Functional Tonotopic Mapping of bVM Neurons

To functionally map the bVM, one optical fiber was implanted at a distinct location within this region in each mouse, with the implantation locations distributed across multiple sites across the bVM when considering the entire cohort. A response trial is defined based on a criterion: a response peak exceeding three times the standard deviation (SD) of baseline within 500 ms after tone onset. BF was defined as the stimulus frequency that elicited the maximum average peak response. To minimize inter-animal variability, the histological sections were aligned to the *Paxinos and Franklin Mouse Brain Atlas* (second edition). The mediolateral coordinate relative to the bregma served as the primary reference for cross-animal alignment. To get the tonotopic map of the bVM, the center point of the optical fiber tip was defined as the recording site coordinate. The horizontal distance from each bVM recording site to the midline was defined as $X_{1}$, and the horizontal span of the bVM was defined as $X_{2}$. The normalized x-coordinate of the bVM ($X_{\text{norm}}$) was calculated as $X_{\text{norm}}=X_{1}/X_{2}$. The vertical distance from each bVM recording site to the midline was defined as $Y_{1}$, and the vertical span of the bVM was defined as $Y_{2}$. The normalized y-coordinate of the bVM ($Y_{\text{norm}}$) was calculated as $Y_{\text{norm}}=Y_{1}/Y_{2}$. BF at each site was visualized using a color map, where normalized mediolateral and dorsoventral positions were plotted along the x- and y-axes, respectively, and BF was color-coded with different colors representing different BF values.

### 2.9 Statistical Analysis

Paired comparisons were analyzed using the two-tailed Wilcoxon signed-rank test, followed by false discovery rate (FDR) correction. Unpaired multi-group comparisons were analyzed using the Kruskal–Wallis test, followed by Dunn’s multiple comparison test. Unpaired two-group comparisons were analyzed using the Mann–Whitney test. A p-value<0.05 was considered statistically significant. All statistical analyses were performed using MATLAB 2021b and GraphPad Prism 8. Data are presented as mean ± standard error of the mean.

## 3 Results

### 3.1 Tonotopic Organization of bVM Neurons Revealed by Fiber Photometry

According to recent studies, distinct subnuclei within the ventromedial thalamus project to specific cortical areas.^44^^,^^45^ To investigate the auditory response properties of bVM neurons that specifically project to A1, we first performed a stereotaxic injection of a retrograde Cre-recombinase expressing AAV (AAV2/2-Retro-hSyn-Cre) into the right A1 [Fig. 1(b)]. This was followed by the injection of a Cre-dependent GCaMP6f virus (AAV2/9-DIO-GCaMP6f) into the ipsilateral bVM. Following a 3-week viral expression, we systematically implanted optical fibers at distinct mediolateral coordinates within the bVM to record pure tone-evoked ${\mathrm{Ca}}^{2+}$ responses of neurons at 26 sites (one recording site per mouse across all animals) using fiber photometry recording [Fig. 1(c)].

### 3.2 Distinct Frequency Preferences of Neurons in Different bVM Subregions

**Fig. 2:**
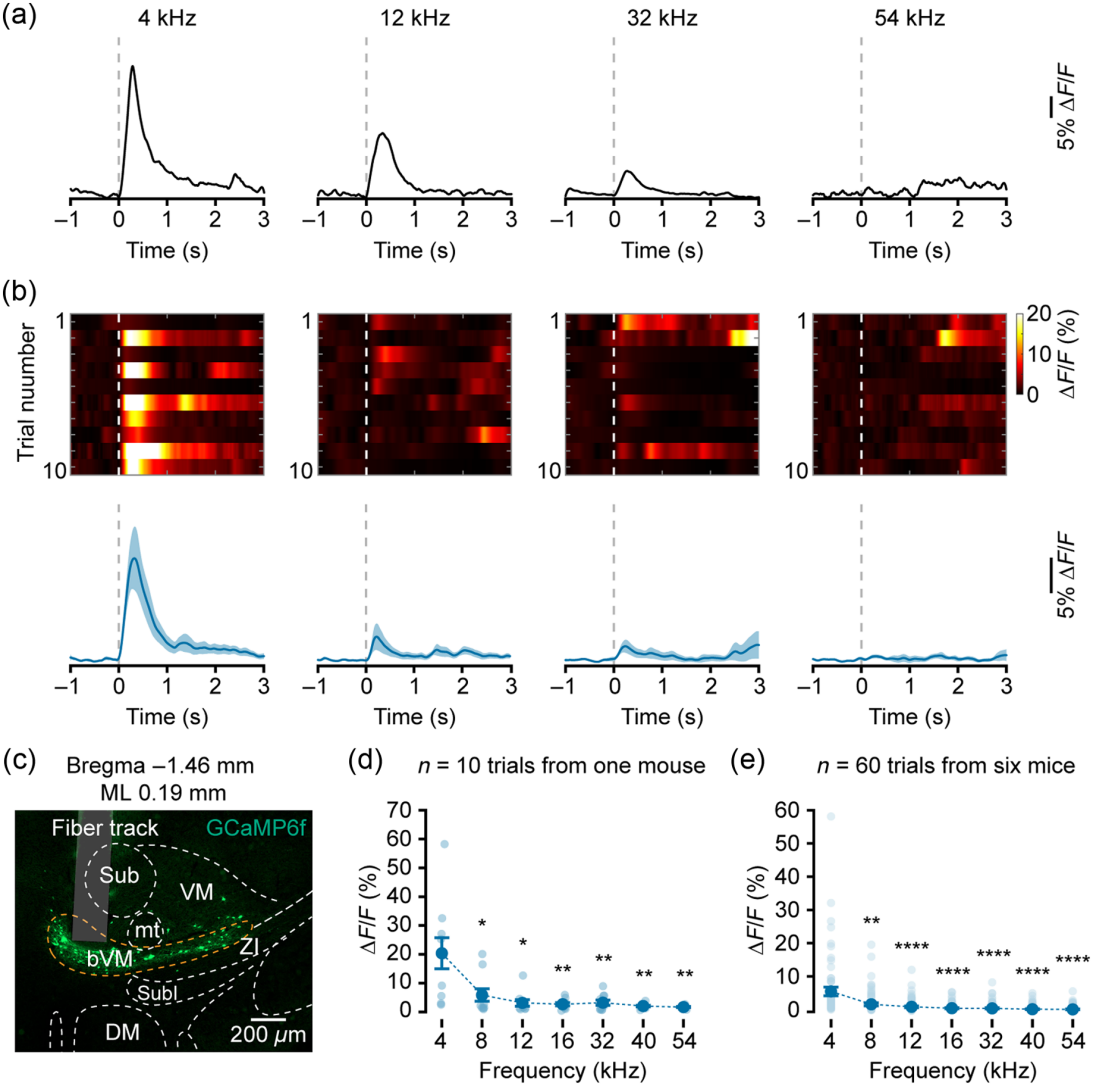
Neuronal responses to pure tones in the low-frequency subregion of the bVM. (a) Representative ${\mathrm{Ca}}^{2+}$ transients evoked by pure tones in a single trial. (b) Top: heatmap of neuronal responses across all trials. Bottom: averaged ${\mathrm{Ca}}^{2+}$ traces across trials. (c) Histological verification of the optical fiber implantation site in the medial bVM. (d) Response amplitudes to different frequencies in the BF = 4 kHz subregion of bVM from one mouse (n=10 trials, *p<0.05, **p<0.01, two-tailed Wilcoxon signed-rank test followed by FDR correction). (e) Response amplitudes to different frequencies in the BF = 4 kHz subregion of bVM across six mice (n=60 trials, **p<0.01, ****p<0.0001, two-tailed Wilcoxon signed-rank test followed by FDR correction).

We first analyzed the recordings along the mediolateral axis of the bVM from individual mice. At a representative medial recording site [Fig. 2(c); AP=−1.46  mm, ML=0.19  mm, and DV=−4.17  mm], the neuronal population exhibited the strongest ${\mathrm{Ca}}^{2+}$ transients in response to pure tone stimuli at a frequency of 4 kHz [Figs. 2(a) and 2(b), bottom], with minimal activation to 54 kHz. The response heat map further showed that robust and consistent activation by 4 kHz was evident across multiple trials [Fig. 2(b), top], forming a clear contrast with responses to other frequencies. Further quantitative analysis of response amplitudes within a 500-ms time window after sound stimulation onset showed that the average peak ${\mathrm{Ca}}^{2+}$ response (ΔF/F) evoked by 4 kHz was significantly larger than most other frequencies [Fig. 2(d); 4 kHz: 20.72±5.06; 8 kHz: 5.08±2.13, 8 kHz versus 4 kHz, p=0.0273; 12 kHz: 3.07±1.07, 12 kHz versus 4 kHz, p=0.0137; 16 kHz: 1.88±0.47, 16 kHz versus 4 kHz, p=0.0039; 32 kHz: 2.56±0.83, 32 kHz versus 4 kHz, p=0.0039; 40 kHz: 1.20±0.30, 40 kHz versus 4 kHz, p=0.0039; and 54 kHz: 0.71±0.16, 54 kHz versus 4 kHz, p=0.0020, two-tailed Wilcoxon signed-rank test followed by FDR correction]. Accordingly, the BF at this site was defined as 4 kHz. Consistent BF=4  kHz tuning properties were observed at medial bVM sites across multiple animals [Fig. 2(e); 4 kHz: 6.11±1.30; 8 kHz: 2.26±0.47, 8 kHz versus 4 kHz, p=0.0023; 12 kHz: 1.51±0.27, 12 kHz versus 4 kHz, $p=3.02\times 10^{-6}$; 16 kHz: 1.10±0.15, 16 kHz versus 4 kHz, $p=2.47\times 10^{-7}$; 32 kHz: 1.05±0.18, 32 kHz versus 4 kHz, $p=1.83\times 10^{-7}$; 40 kHz: 0.80±0.13, 40 kHz versus 4 kHz, $p=2.24\times 10^{-7}$; and 54 kHz: 0.75±0.12, 54 kHz versus 4 kHz, $p=2.99\times 10^{-7}$, two-tailed Wilcoxon signed-rank test followed by FDR correction].

**Fig. 3:**
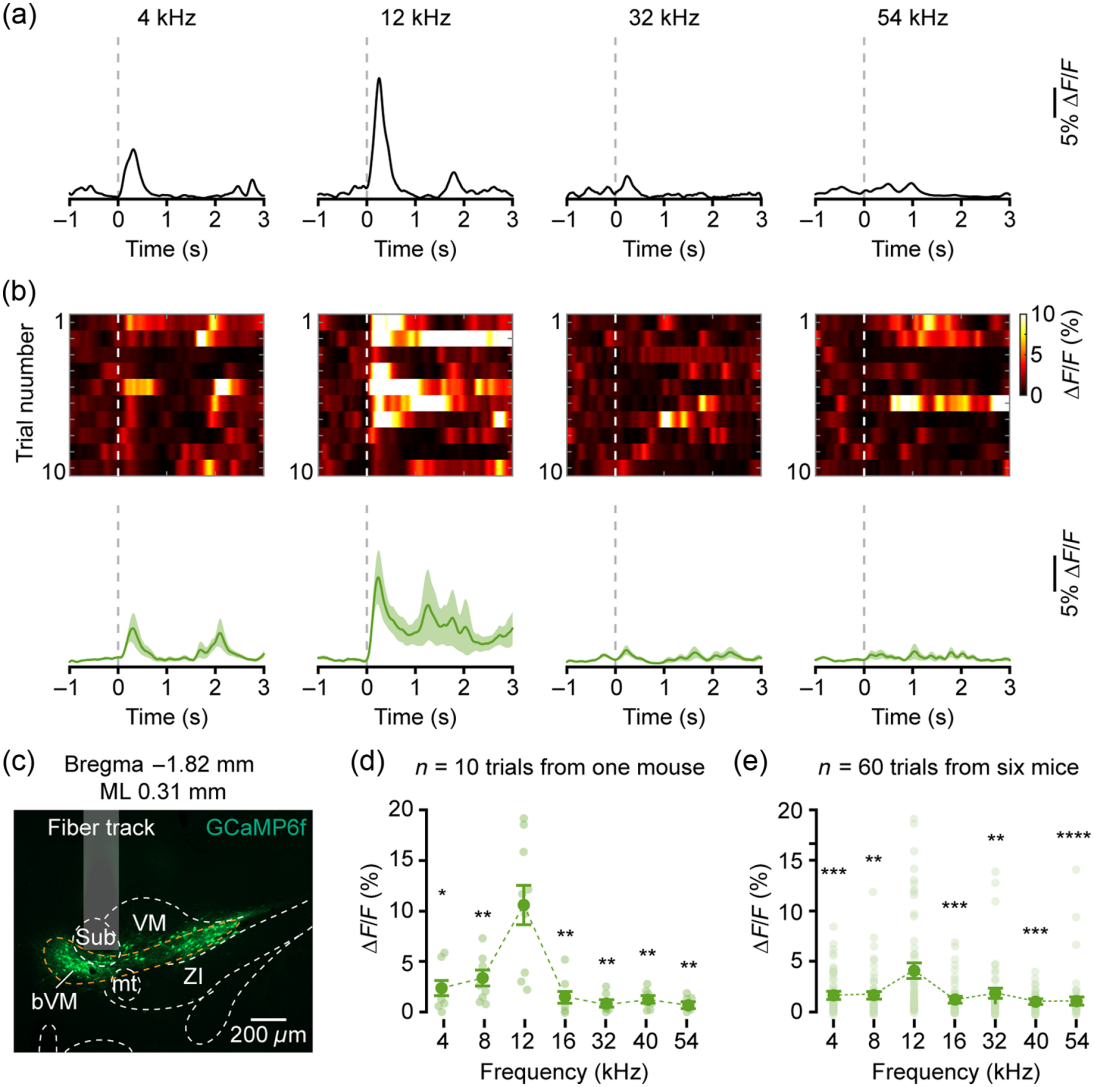
Neuronal responses to pure tones in the mid-frequency subregion of the bVM. (a) Representative ${\mathrm{Ca}}^{2+}$ transients evoked by pure tones in a single trial. (b) Top: response heatmap across all trials. Bottom: trial-averaged ${\mathrm{Ca}}^{2+}$ traces. (c) Histological verification of the fiber track location in the bVM. (d) Response amplitude to different frequencies in the BF = 12 kHz subregion of bVM from one mouse (n=10 trials, *p<0.05, **p<0.01, two-tailed Wilcoxon signed-rank test followed by FDR correction). (e) Response amplitude to different frequencies in the BF = 12 kHz subregion of bVM across six mice (n=60 trials, **p<0.01, ***p<0.001, ****p<0.0001, two-tailed Wilcoxon signed-rank test followed by FDR correction).

Recordings at a mediolateral coordinate of 0.31 mm from the midline [Fig. 3(c); AP=−1.82  mm, ML = 0.31 mm, and DV=−4.21  mm] revealed distinct tuning properties. At this site, the neuronal population exhibited maximal ${\mathrm{Ca}}^{2+}$ responses to 12 kHz [Figs. 3(a) and 3(b), bottom]. Lower frequencies also evoked responses in these neurons (though of lesser magnitude than those to 12 kHz), whereas responses to high-frequency tones were minimal. The response heat map further showed consistent robust activation by 12 kHz across multiple trials [Fig. 3(b), top], indicating a clear preference of neurons at this site for 12 kHz. Quantitative analysis of response amplitudes within a 500-ms time window following tone onset confirmed that the average peak Ca2+ response (ΔF/F) at 12 kHz was significantly larger than that at most other tested frequencies [Fig. 3(d)], establishing 12 kHz as the BF for this site (4 kHz: 2.29±0.62, 4 kHz versus 12 kHz, p=0.0117; 8 kHz: 3.33±0.69, 8 kHz versus 12 kHz, p=0.0059; 12 kHz: 10.57±1.95; 16 kHz: 1.43±0.48, 16 kHz versus 12 kHz, p=0.0098; 32 kHz: 0.82±0.25, 32 kHz versus 12 kHz, p=0.0039; 40 kHz: 1.22±0.25, 40 kHz versus 12 kHz, p=0.0029; and 54 kHz: 0.64±0.19, 54 kHz versus 12 kHz, p=0.0023, two-tailed Wilcoxon signed-rank test followed by FDR correction). Analysis of tone-evoked response across multiple animals consistently identified 12 kHz as the BF for this subregion [Fig. 3(e); 4 kHz: 1.59±0.26, 4 kHz versus 12 kHz, p=0.0003; 8 kHz: 1.60±0.28, 8 kHz versus 12 kHz, p=0.0019; 12 kHz: 4.07±0.65; 16 kHz: 1.18±0.20, 16 kHz versus 12 kHz, p=0.0003; 32 kHz: 1.80±0.35, 32 kHz versus 12 kHz, p=0.0039; 40 kHz: 1.0±0.13, 40 kHz versus 12 kHz, p=0.0003; and 54 kHz: 1.20±0.26, 54 kHz versus 12 kHz, $p=7.11\times 10^{-5}$, two-tailed Wilcoxon signed-rank test followed by FDR correction].

**Fig. 4:**
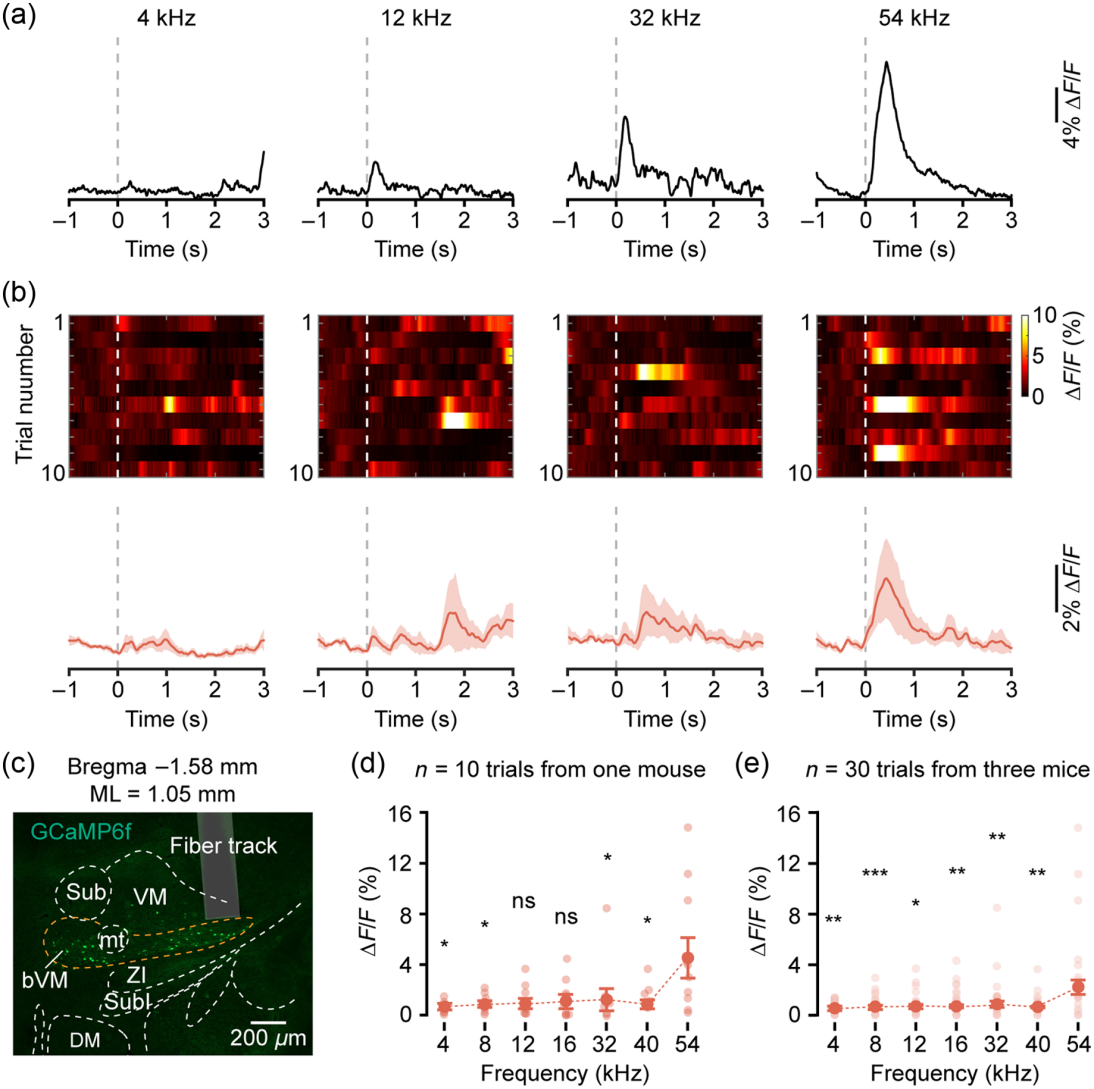
Neuronal responses to pure tones in the high-frequency subregion of the bVM. (a) Representative ${\mathrm{Ca}}^{2+}$ transients evoked by pure tones in a single trial. (b) Top: response heatmap across all trials. Bottom: trial-averaged ${\mathrm{Ca}}^{2+}$ traces. (c) Histological verification of the fiber implantation site in the lateral bVM. (d) Response amplitude to different frequencies in the BF = 54 kHz subregion of bVM from one mouse (n=10 trials, *p<0.05, ns: not significant, two-tailed Wilcoxon signed-rank test followed by FDR correction). (e) Response amplitude to different frequencies in the BF = 54 kHz subregion of bVM across three mice (n=30 trials, *p<0.05, **p<0.01, ***p<0.001, two-tailed Wilcoxon signed-rank test followed by FDR correction).

Extending our recordings to the lateral subregion of the bVM [Fig. 4(c); AP=−1.58  mm, ML = 1.05 mm, and DV=−3.96  mm], we observed a preference for high-frequency tones, with robust responses to 54 kHz [Figs. 4(a) and 4(b), bottom]. In contrast, responses to mid-frequency tones were comparatively weak, and minimal responses were detected to low-frequency stimuli. The response heat map further confirmed that the robust activation by 54 kHz occurred consistently across multiple trials [Fig. 4(b), top], reinforcing the frequency-specific preference of this subregion. Quantitative analysis of response amplitudes within the 500-ms time window following tone onset demonstrated that the average peak response (ΔF/F) at 54 kHz was significantly larger than that at most other frequencies, establishing 54 kHz as the BF for this lateral site [Fig. 4(d); 4 kHz: 0.70±0.20, 4 kHz versus 54 kHz, p=0.0234; 8 kHz: 0.86±0.25, 8 kHz versus 54 kHz, p=0.0313; 12 kHz: 0.90±0.40, 12 kHz versus 54 kHz, p=0.0938; 16 kHz: 1.08±0.49, 16 kHz versus 54 kHz, p=0.1953; 32 kHz: 1.13±0.83, 32 kHz versus 54 kHz, p=0.0234; and 40 kHz: 0.83±0.33, 40 kHz versus 54 kHz, p=0.0351; 54 kHz: 4.95±1.58, two-tailed Wilcoxon signed-rank test followed by FDR correction]. Consistent with this finding, analysis of tone-evoked responses across multiple animals identified 54 kHz as the BF for this lateral subregion [Fig. 4(e); 4 kHz: 0.54±0.09, 4 kHz versus 54 kHz, p=0.0028; 8 kHz: 0.62±0.12, 8 kHz versus 54 kHz, p=0.0006; 12 kHz: 0.68±0.15, 12 kHz versus 54 kHz, p=0.033; 16 kHz: 0.68±0.17, 16 kHz versus 54 kHz, p=0.006; 32 kHz: 0.80±0.25, 32 kHz versus 54 kHz, p=0.002; and 40 kHz: 0.61±0.11, 40 kHz versus 54 kHz, p=0.0012; 54 kHz: 2.17±0.56, two-tailed Wilcoxon signed-rank test followed by FDR correction].

Together, these results indicate a systematic progression in frequency tuning along the mediolateral axis of the bVM, from low frequencies medially to high frequencies laterally.

### 3.3 Frequency Responses of bVM Neurons at Different Sound Pressure Levels

**Fig. 5:**
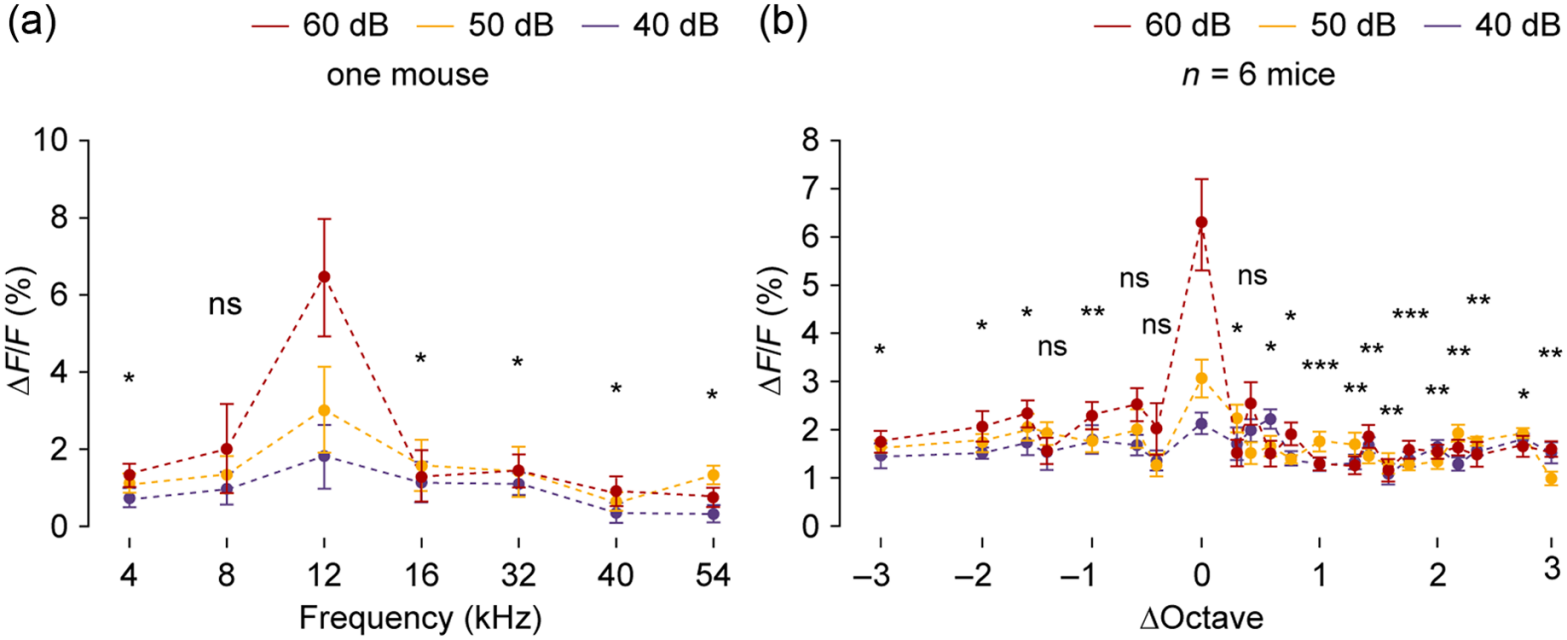
Pure-tone evoked neuronal responses across bVM subregions at varying SPLs. (a) Average response amplitudes of bVM neurons to pure tones at 40-, 50-, and 60-dB SPL in a representative mouse (n=10 trials from one mouse, *p<0.05, ns: not significant, two-tailed Wilcoxon signed-rank test followed by FDR correction). (b) Average response amplitudes of bVM neurons to pure tones at 40-, 50-, and 60-dB SPL across six mice (n=6 mice, *p<0.05, **p<0.01, ***p<0.001, ns: not significant, two-tailed Wilcoxon signed-rank test followed by FDR correction).

As frequency tuning can depend on sound intensity,^18^ we systematically characterized bVM neuronal responses to pure tones at three sound pressure levels (40-, 50-, and 60-dB SPL). In a representative animal exhibiting a best frequency of 12 kHz, frequency selectivity was intensity-dependent [Fig. 5(a)]. At 60-dB SPL, neurons exhibited sharp and unambiguous frequency selectivity, with 12 kHz evoking the largest responses (4 kHz: 1.33±0.30, 4 kHz versus 12 kHz, p=0.0312; 8 kHz: 2.02±1.14, 8 kHz versus 12 kHz, p=0.1094; 12 kHz: 6.45±1.52; 16 kHz: 1.31±0.66, 16 kHz versus 12 kHz, p=0.0469; 32 kHz: 1.44±0.43, 32 kHz versus 12 kHz, p=0.0312; 40 kHz: 0.93±0.38, 40 kHz versus 12 kHz, p=0.0117; and 54 kHz: 0.74±0.20, 54 kHz versus 12 kHz, p=0.0156, two-tailed Wilcoxon signed-rank test followed by FDR correction). Although emerging trends of frequency selectivity were observed at 40- and 50-dB SPL [Fig. 5(a); Tables S1 and S2 in the Supplementary Material], response amplitude differences across most frequency pairs did not reach statistical significance after multiple comparisons correction. Population analysis across six mice confirmed that frequency selectivity was most robust at 60-dB SPL [Fig. 5(b); Table S3 in the Supplementary Material], whereas reduced selectivity was observed at lower intensities [Fig. 5(b); Tables S4 and S5 in the Supplementary Material]. Within each SPL condition, we compared neural responses at the reference frequency (octave = 0, corresponding to the best frequency) against all other frequency offsets. Based on these findings, all subsequent quantitative analyses were performed using pure tone stimuli at 60-dB SPL to ensure robust frequency-specific responses.

### 3.4 Pure Tone-Evoked Response Properties Across the bVM

**Fig. 6:**
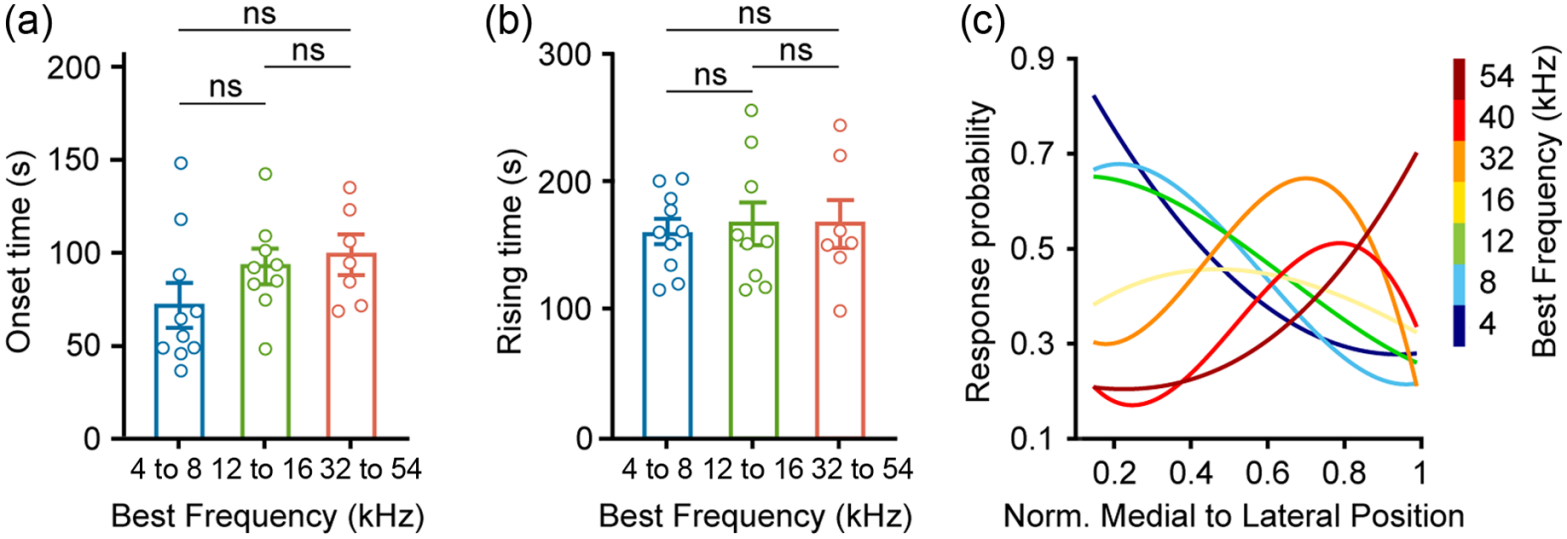
Pure-tone response properties of neurons across different locations in the bVM. (a) and (b) Comparison of neuronal response onset time (a) and rising time (b) among three BF ranges (4 to 8 kHz: n=10 mice, 12 to 16 kHz: n=9 mice, and 32 to 54 kHz: n=7 mice, ns: not significant, Kruskal–Wallis test followed by Dunn’s multiple comparisons test). (c) Polynomial curve fitting of response probability in bVM neurons to pure tones across different locations.

We next examined whether the temporal dynamics of tone-evoked Ca2+ responses in bVM neurons varied systematically with their frequency preference. Response onset time was defined as the time from tone onset to 10% of peak amplitude, and rising time as the duration between 10% and 90% of peak amplitude.^46^ Neuron populations were grouped by their BF range [Figs. 6(a) and 6(b)]. When comparing onset time or rising time among low-, mid-, and high-BF neuron groups (low-BF: 4 to 8 kHz; mid-BF: 12 to 16 kHz; and high-BF: 32 to 54 kHz), no significant differences were observed among the three groups (p>0.05, Kruskal–Wallis test followed by Dunn’s multiple comparisons test). We next analyzed the response probability across recording locations by polynomial fitting (see Sec. 2). A trial was considered responsive when the peak response exceeded three times the SD of baseline.^36^^,^^47^ Polynomial fits of response probability across 26 sites showed higher probabilities for low frequencies at medial sites and for high frequencies at lateral sites [Fig. 6(c)]. This spatial gradient in response probability provided complementary evidence for the frequency-specific organization of bVM.

### 3.5 Correlation Between Best Frequency and Mediolateral Spatial Distribution in bVM Neurons

**Fig. 7:**
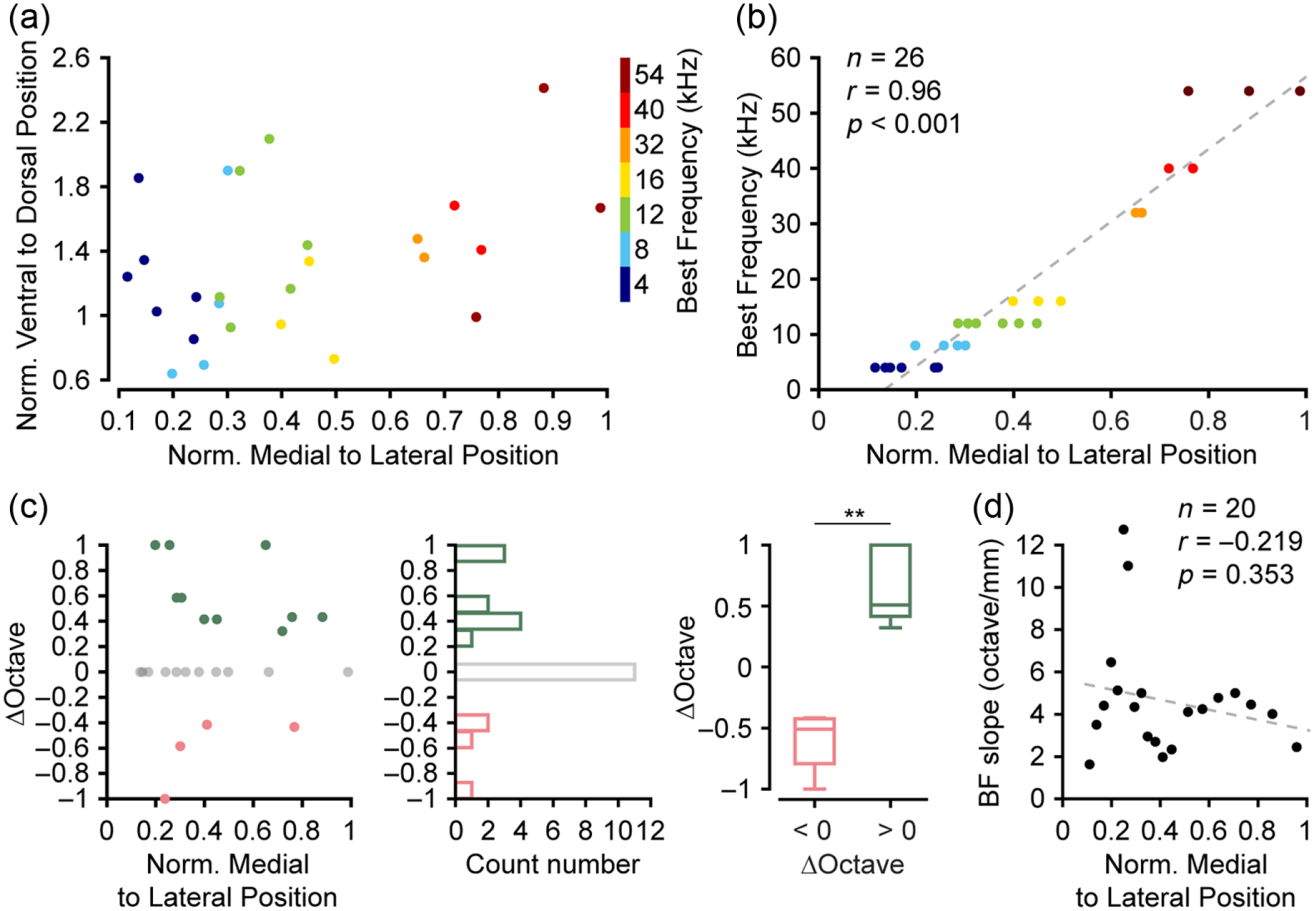
Tonotopic organization of bVM neurons. (a) Spatial distribution of recording sites in the bVM of a representative mouse. Circle color indicates the BF of each site. (b) Linear correlation between normalized recording site position and BF, confirming a tonotopic gradient across the bVM. Circle color indicates the BF of each site, consistent with panel (a) (n=26 mice, r=0.96, ***p<0.001, Pearson’s correlation). (c) Left: scatter plot of frequency differences among adjacent recording sites across the bVM. BF shift was defined as the BF difference between adjacent lateral and current recording sites (positive: higher BF laterally; negative: lower BF laterally; zero: no change). Middle: histogram shows the distribution of the BF shift. Right: comparison of positive and negative octave-scale shifts (**p<0.01, Mann–Whitney test, octave < 0 group: n=4 mice, octave > 0 group: n=10 mice). (d) Slope of BF changes along the medial-to-lateral axis in the bVM (n=20 mice, r=−0.219, p=0.353, Pearson’s correlation).

To visualize the tonotopic organization across the bVM, we constructed a spatial frequency map based on BF data from all 26 histologically verified recording sites [Fig. 7(a)]. The normalized mediolateral and ventrodorsal coordinates of each site defined its location on this map, and its BF was then represented by a color-coded circle. This visualization revealed a clear tonotopic gradient along the mediolateral axis of the bVM. Linear regression and Pearson correlation analysis confirmed a significant positive correlation between normalized position and BF [Fig. 7(b); Pearson correlation, r=0.96, p<0.001, n=26 mice]. We further examined the local structure by analyzing the BF shift among adjacent recording sites [Fig. 7(c)]. This analysis showed that some sites had BFs higher than their more medial neighbors. Sites with a positive shift were more common than those with a negative shift (p=0.002, Mann–Whitney test). Finally, we analyzed the slope of BF changes [Fig. 7(d)]. As a seven-site window was used to calculate the slope (see Sec. 2), the number of mice here is 20. The slope of change at all points was positive, indicating an increase in frequency preference from medial to lateral bVM. Although the fitted line of the BF change slope showed a decreasing trend, this trend was not statistically significant, indicating there is no reliable evidence for an overrepresentation of specific frequencies within the bVM (Pearson correlation, r=−0.219, p=0.353, n=20 mice). Collectively, these results confirm that the bVM exhibits a robust, spatially ordered tonotopic map.

## 4 Discussion and Conclusion

Although the tonotopic organization of the classical auditory pathway is well established, systematic investigation of the higher-order thalamic nuclei involved in fine auditory discrimination remains limited. In this study, we combined Cre-dependent retrograde viral labeling with fiber photometry to characterize the response properties of bVM neurons projecting to A1 in response to pure tone stimuli. We uncovered a robust mediolateral tonotopic gradient: the neurons in the medial bVM preferred low frequencies, whereas those in the lateral bVM preferred high frequencies. This spatial–functional organization was strongly supported by a significant correlation between anatomical position and BF, consistent with our recent anatomical findings.^26^ Moreover, bVM neurons exhibited frequency-selective enhancement in both response amplitude and probability, indicating enhanced reliability in coding of preferred frequencies. These results provide direct functional evidence for a tonotopic organization within the bVM, substantiating and extending our prior anatomical observations.

The tonotopic organization of bVM neurons aligns with the functional architecture of the classical auditory pathway,^18^ where frequency selectivity serves as a fundamental organizing principle, and BF varies systematically along spatial gradients. However, the bVM is not merely a parallel relay to the MGBv for conveying tonotopically organized information to the AuC. Instead, its tonotopic organization may serve distinct functional roles compared with the MGBv. Unlike the MGBv, which densely projects to layer IV of the AuC and acts as a primary “driver” of feedforward sensory information flow,^13^ the bVM primarily targets neuron-derived neurotrophic-factor-positive interneurons (NDNF-INs) in cortical layer 1.^26^ Existing studies suggest that NDNF-INs integrate top-down modulatory inputs and participate in brain state regulation.^48^ Notably, NDNF-INs also receive inputs from the higher-order medial geniculate (HO-MG).^49^ The HO-MG pathway lacks clear tonotopic organization and is more involved in complex sound processing and cognitive integration. In contrast, the bVM provides frequency-tuned input, and its frequency selectivity can be dynamically modified through auditory learning.^26^ This positions the bVM as a critical node bridging classical sensory relay and higher-order integration.

From a systems neuroscience perspective, the existence of hierarchical tonotopic maps reflects a core principle of neural computation. Spatially organized mappings optimize information processing by minimizing wiring redundancy, enabling parallel computation, and providing a stable structural framework for experience-dependent plasticity.^50^ Consequently, the maintenance of hierarchical tonotopic representations across the thalamus and auditory cortex may enable parallel processing streams for high-fidelity acoustic feature extraction and behaviorally relevant signal filtering. Building on this framework, we propose that A1 and bVM represent complementary processing pathways, whereas A1 is dedicated to generating high-fidelity spectrotemporal representations that precisely encode the physical sound attributes (e.g., frequency, intensity, and temporal dynamics) for fine-grained acoustic analysis,^8^ the bVM integrates specific frequency information with behavioral state signals (e.g., attention and learning) that originate from upstream regions including the thalamic reticular nucleus (TRN) and the prelimbic prefrontal cortex.^26^ Through its projections to layer 1 NDNF-INs of the AuC, the bVM may exert frequency-selective gain control over cortical circuits. Analogous to how the pulvinar in the visual system preserves retinotopic organization to enable spatially specific attentional modulation,^51^^,^^52^ the frequency-specific gain modulation mediated by the bVM may constitute a neural substrate for auditory stream segregation and selective attention. This hypothesis could be tested in future studies by selectively manipulating bVM activity during fine discrimination tasks while monitoring frequency-specific changes in cortical gain.

Notably, such higher-order auditory processing is not confined to a single thalamic nucleus. Growing evidence shows that the auditory thalamus MGB encodes learning-related information and behavioral choices during auditory tasks.^53^^,^^54^ Other thalamic nuclei beyond the auditory pathway also play important roles: for example, the mediodorsal thalamus (MD) contributes to auditory-induced arousal from slow-wave sleep,^55^ whereas the TRN acts as a dynamic gatekeeper that selectively regulates thalamocortical auditory transmission based on behavioral context.^56^ Together, these findings indicate that higher-order auditory functions rely on the combined contributions of multiple thalamic nuclei and reciprocal corticothalamic loops,^57^ forming a distributed network that balances faithful sensory transmission with dynamic, context-dependent modulation essential for adaptive auditory processing.

Another important question is how the frequency specificity in bVM is established. Our previous retrograde tracing and chemogenetic experiments identified the inferior colliculus (IC, including the CIC) as a major auditory input to the bVM.^26^ Given that the CIC possesses a well-defined tonotopic map,^17^^,^^27^ it is highly probable that the bVM receives tonotopic inputs from the CIC.

Methodologically, we selected a stimulus intensity of 60-dB SPL to reconstruct the bVM tonotopic map. This intensity provided an optimal balance, eliciting robust frequency-selective responses while minimizing the occurrence of bimodal tuning profiles. Beyond establishing this map, our study demonstrates the utility of fiber photometry as a simple yet efficient strategy for mapping population-level activity in deep brain structures. Its efficacy in auditory research is further supported by recent studies showing frequency-specific population activity in subcortical regions during tasks such as fear conditioning.^58^^,^^59^ Although those studies did not explicitly map tonotopy, they support the utility of fiber photometry for resolving frequency-specific activity.

Our approach has several limitations that should be noted. First, although fiber photometry effectively captures population dynamics in deep brain regions, it lacks the resolution to determine single-neuron tuning properties. Second, the physical diameter of the optical fiber (200  μm) limited the spatial sampling density within individual animals. We mitigated this constraint by adopting a “one recording site per animal” strategy across a relatively large cohort (n=26 mice). This approach allowed us to successfully delineate the macroscopic tonotopic organization, which is fundamentally a population-level property. Future studies will require next-generation two-photon imaging or advanced optrode systems capable of overcoming the current limitations for mapping deep brain regions such as the bVM. Such technological advances would help us to refine the tonotopic map of the bVM at cellular resolution and characterize precise temporal response features.

In conclusion, our study provides functional evidence that the bVM possesses a spatially organized tonotopic map. When considered alongside its distinct projection target in cortical layer 1, this organization suggests that the bVM plays a critical role in shaping fine-grained auditory perception and behavior. Future research should investigate the functional dynamics of this bVM map under behavior-related listening conditions and explore how it is modulated by attention, learning, and internal states.

## Supplementary Material

10.1117/1.NPh.13.1.015008.s01

## Data Availability

The data presented in this study are available from the corresponding author upon reasonable request.
