# Supplementary material for: Tonotopic organization in the basal region of the ventromedial nucleus of the thalamus revealed by fiber photometry recording
Source: Neurophotonics. 2026 Jan 29;13(1):015008. doi: 10.1117/1.NPh.13.1.015008 (PMC12853032; doi:10.1117/1.NPh.13.1.015008)
Supplement: Supplementary file 1 [file NPh_013_015008_SD001.pdf]

Supplementary Material:

Table S1 Statistical analysis results of bVM neuronal response to pure tone at 40 dB SPL in Fig.5(a)

Frequency (kHz)	Mean ΔF/F	SEM	P value after FDR correction
4	0.76	0.25	0.1953
8	0.97	0.38	0.9453
12	1.82	0.82	
16	1.15	0.51	1.0
32	1.12	0.28	0.46
40	0.37	0.23	0.38
54	0.34	0.22	0.1484

Table S2 Statistical analysis results of bVM neuronal response to pure tone at 50 dB SPL in Fig.5(a)

Frequency (kHz)	Mean ΔF/F	SEM	P value after FDR correction
4	1.15	0.26	0.0156
8	1.36	0.50	0.0469
12	3.01	1.10	
16	1.60	0.66	0.8125
32	1.43	0.65	0.9375
40	0.63	0.31	0.0625
54	1.35	0.23	0.0625

Table S3 Statistical analysis results of bVM neuronal response to pure tone at 40 dB SPL in Fig.5(b)

Octave	Mean ΔF/F	SEM	P value after FDR correction
-3	1.46	0.22	0.6861
-2	1.52	0.12	0.8729
-1.58	1.81	0.22	0.6670
-1.42	1.62	0.39	0.9570
-1	1.84	0.25	0.8614
-0.58	1.76	0.14	0.8615
-0.42	1.35	0.14	0.6878
0	2.26	0.19	

0.32	1.79	0.34	0.6387
0.42	2.04	0.22	0.9264
0.58	2.22	0.17	0.1641
0.75	1.41	0.15	0.9067
1	1.28	0.10	0.0835
1.32	1.33	0.15	0.9102
1.42	1.76	0.17	0.8306
1.58	1.17	0.21	0.5961
1.75	1.47	0.13	0.4539
2	1.64	0.14	0.5706
2.17	1.29	0.14	0.7475
2.32	1.57	0.10	0.8531
2.75	1.81	0.11	0.3828
3	1.52	0.22	0.7045

Table S4 Statistical analysis results of bVM neuronal response to pure tone at 50 dB SPL in Fig.5(b)

Octave	Mean ΔF/F	SEM	P value after FDR correction
-3	1.65	0.13	0.0570
-2	1.77	0.14	0.012
-1.58	2.14	0.24	0.0591
-1.42	1.93	0.18	0.2030
-1	1.78	0.19	0.0104
-0.58	2.01	0.36	0.0387
-0.42	1.28	0.20	0.0410
0	3.15	0.35	
0.32	2.24	0.25	0.5566
0.42	1.69	0.20	0.0119
0.58	1.83	0.21	0.0540
0.75	1.39	0.09	0.0117
1	1.76	0.17	0.0108
1.32	1.70	0.13	0.0487
1.42	1.54	0.13	0.0120
1.58	1.38	0.20	0.0103
1.75	1.37	0.09	0.0002
2	1.34	0.16	0.0139
2.17	1.93	0.17	0.0273
2.32	1.76	0.10	0.0261
2.75	1.93	0.10	0.0239
3	0.99	0.14	0.0082

Table S5 Statistical analysis results of bVM neuronal response to pure tone at 60 dB SPL in Fig.5(b)

Octave	Mean ΔF/F	SEM	P value after FDR correction
-3	1.75	0.19	0.0293
-2	2.15	0.28	0.0171
-1.58	2.23	0.25	0.0222
-1.42	1.64	0.24	0.0570
-1	2.29	0.29	0.0019
-0.58	2.50	0.30	0.0877
-0.42	2.07	0.56	0.1309
0	6.34	0.95	
0.32	1.61	0.25	0.0239
0.42	2.63	0.44	0.0976
0.58	1.59	0.25	0.0273
0.75	1.91	0.20	0.0256
1	1.29	0.10	0.0001
1.32	1.26	0.16	0.0091
1.42	1.94	0.20	0.0058
1.58	1.25	0.21	0.0068
1.75	1.67	0.14	0.0005
2	1.54	0.18	0.0021
2.17	1.63	0.16	0.0020
2.32	1.49	0.26	0.0059
2.75	1.66	0.22	0.0338
3	1.60	0.16	0.0082
